# Diagnostic and referral pathways in patients with rare lipodystrophy and insulin-resistance syndromes: key milestones assessed from a national reference center

**DOI:** 10.1186/s13023-024-03173-2

**Published:** 2024-04-27

**Authors:** Bruno Donadille, Sonja Janmaat, Héléna Mosbah, Inès Belalem, Sophie Lamothe, Mariana Nedelcu, Anne-Sophie Jannot, Sophie Christin-Maitre, Bruno Fève, Camille Vatier, Corinne Vigouroux

**Affiliations:** 1 Saint-Antoine Hospital, Reference Center for Rare Diseases of Insulin Secretion and Insulin Sensitivity (PRISIS), Department of Endocrinology, Assistance Publique-Hôpitaux de Paris (AP-HP), 184 rue du Faubourg Saint-Antoine, 75012 Paris, France; 2 Saint-Antoine Research Center, Institute of CardioMetabolism and Nutrition (ICAN), Sorbonne University, Inserm UMR_S 938, Paris, France; 3 Banque Nationale de Données Maladies Rares, DSN-I&D, APHP, Paris, France; 4 Sorbonne Université, Inserm UMR_S 933, Paris, France

**Keywords:** Lipodystrophy, Insulin-resistance, Diagnosis, Age, Rare diseases, National health database, Diagnostic delay, Reference center, Referral

## Abstract

**Background:**

Rare syndromes of lipodystrophy and insulin-resistance display heterogeneous clinical expressions. Their early recognition, diagnosis and management are required to avoid long-term complications.

**Objective:**

We aimed to evaluate the patients’ age at referral to our dedicated national reference center in France and their elapsed time from first symptoms to diagnosis and access to specialized care.

**Patients and methods:**

We analyzed data from patients with rare lipodystrophy and insulin-resistance syndromes referred to the coordinating PRISIS reference center (Adult Endocrine Department, Saint-Antoine Hospital, AP-HP, Paris), prospectively recorded between 2018 and 2023 in the French National Rare Disease Database (BNDMR, *Banque Nationale de Données Maladies Rares*).

**Results:**

A cohort of 292 patients was analyzed, including 208 women, with the following diagnosis: Familial Partial LipoDystrophy (FPLD, *n* = 124, including *n* = 67 FPLD2/Dunnigan Syndrome); Acquired lipodystrophy syndromes (*n* = 98, with *n* = 13 Acquired Generalized Lipodystrophy, AGL); Symmetric cervical adenolipomatosis (*n* = 27, Launois-Bensaude syndrome, LB), Congenital generalized lipodystrophy (*n* = 18, CGL) and other rare severe insulin-resistance syndromes (*n* = 25). The median age at referral was 47.6 years [IQR: 31–60], ranging from 25.2 (CGL) to 62.2 years old (LB). The median age at first symptoms of 27.6 years old [IQR: 16.8–42.0]) and the median diagnostic delay of 6.4 years [IQR: 1.3–19.5] varied among diagnostic groups. The gender-specific expression of lipodystrophy is well-illustrated in the FPLD2 group (91% of women), presenting with first signs at 19.3 years [IQR: 14.4–27.8] with a diagnostic delay of 10.5 years [IQR: 1.8–27.0].

**Conclusion:**

The national rare disease database provides an important tool for assessment of care pathways in patients with lipodystrophy and rare insulin-resistance syndromes in France. Improving knowledge to reduce diagnostic delay is an important objective of the PRISIS reference center.

## Introduction

Lipodystrophy syndromes are heterogeneous chronic rare diseases, characterized by anatomical and functional defects of adipose tissue resulting from genetic or acquired causes. Lipodystrophy syndromes present as systemic diseases with progressive metabolic alterations associated with insulin-resistance, such as diabetes, hypertriglyceridemia, non-alcoholic fatty liver disease, and ovarian dysfunction in women, with multi-tissue involvement depending on the underlying cause. Besides different clinical presentations with generalized or partial lipoatrophy, with or without fat overgrowth in body areas without lipoatrophy, these syndromes are highly heterogeneous diseases. The onset of lipodystrophy may be precocious, from birth or early infancy, or delayed in late childhood or adulthood. Some subtypes of lipodystrophy are associated with specific clinical signs and complications, such as cardiovascular and/or neurological involvement [1–3]. Acquired forms of lipodystrophies generally result from autoimmune mechanisms and/or iatrogenic therapies. The identification of causative pathogenic variants in more than 20 genes leading to monogenic forms of lipodystrophies has highlighted several determinants in adipose tissue pathophysiology. Molecular and cellular bases of monogenic lipodystrophy syndromes involve, among others, altered adipocyte differentiation, structure and/or regulation of the adipocyte lipid droplet and/or premature cellular senescence. This field of research, still highly productive, indicates adipose tissue as a major actor to ensure proper whole-body insulin sensitivity [4]. Other rare conditions characterized by severe insulin-resistance without primary adipose tissue dysfunction result from direct defects in insulin signaling pathways, due to specific genetic or dysimmune disorders [5]. The four main forms of Congenital Generalized Lipodystrophy, referred to as CGL1 to CGL4, are due to pathogenic variants in *AGPAT2*, *BSCL2*, *CAV1*, and *CAVIN1*/*PTRF* genes, respectively. Regarding Familial Partial Lipodystrophy (FPLD), the main subgroups, FPLD2 to 6, are associated with pathogenic variants in *LMNA*, *PPARG*, *PLIN1*, *CIDEC* and *LIPE* respectively, whereas FPLD1 is thought to be of polygenic origin [6, 7].

Diagnosis of lipodystrophy and rare insulin-resistance syndromes is based on clinical, metabolic, and genetic investigations. It is important to note that underdiagnosis of lipodystrophy syndromes is probably widespread, as indicated by the major discrepancies in their estimated prevalence, ranging from 3 cases/million to 1 in 20,000 individuals [8, 9], a higher prevalence being reported in genetic isolates, due to a founder effect [10, 11]. However, timely diagnosis is key to optimize specialized medical care, to establish the required multidisciplinary approach for patient disease management, to provide genetic counselling and avoid complications, particularly at the metabolic/cardiovascular levels [6].

In France, the main objectives of the third national plan for rare diseases launched in 2017 are to improve medical care, to provide equal access to healthcare in patients with rare diseases [12, 13], to decrease diagnostic delay, to reinforce care coordination and to elevate knowledge of non-specialist caregivers. The PRISIS national reference (Rare Diseases of Insulin Secretion and Insulin Sensitivity, namely, for *Pathologies Rares de l’Insulino-Sécrétion et de l’Insulino-Sensibilité)* network was created in December 2017 under this government plan, within the French healthcare network for rare endocrine disease (namely, FIRENDO, for *Filière Maladies Rares Endocriniennes*). Today, PRISIS comprises one coordinating adult reference center, two constitutive reference centers and 24 competence centers spread across the country. Patients referred to rare disease centers in France are recorded through the BaMaRa information system [14], which collects a minimum dataset on epidemiology and natural history of rare diseases. for all patients attending rare disease centers accredited by the French Ministry of Health [15]. These data are further deidentified and stored in the French National Rare Disease Database (BNDMR; namely “*Banque Nationale de Données Maladies Rares*”).

French national guidelines recommend that patients with lipodystrophy should be referred to a PRISIS competence or reference center to organize initial management and follow-up [16].

We took advantage of data implemented by the Paris-Saint-Antoine PRISIS reference center in the BNDMR to evaluate the age at onset of clinical symptoms, the diagnostic delay, and the referral time frame in adult patients with lipodystrophy and insulin-resistance syndromes.

## Patients and methods

### Patients

This study included all patients with a suspected or confirmed diagnosis of rare lipodystrophy and/or insulin-resistance syndromes referred to the PRISIS coordinating reference center, located in the adult endocrinology department of Saint-Antoine Hospital, Paris, France, and registered in the French National Rare Disease Database (BNDMR) from 1st January 2018 to 31 August 2023. The BaMaRa-BNDMR infrastructure was set up in accordance to national ethics requirements [14], the study was approved by the BNDMR scientific committee (see specific section below). All patients included in the BNDMR database have provided their consent to the re-use their BNDMR data for research purposes.

### Study protocol

We extracted from the BNDMR the local data related to patients who were referred to Saint-Antoine Hospital from 1st January 2018 to 31 August 2023. We recorded the following parameters: date of birth, age at last follow-up, sex, distance to reference center, origin of the referral (General Practitioner (GP), pediatrician, adult specialist, through patient initiative), age at referral, as well as the age at first clinical sign and at diagnosis when available. The *diagnosis delay* was defined as the elapsed time between the age at first clinical signs of the disease and the age at diagnosis as declared by the physician in the database. The *referral delay* was defined as the elapsed time between the age at first clinical signs and the age at first referral in our center.

### Diagnostic classification

Diagnosis of lipodystrophy and rare extreme insulin-resistance syndromes is based on clinical, biochemical, imaging and/or molecular diagnosis. The diagnosis of diseases with a known genetic cause was confirmed by molecular analysis. Patients were diagnosed with one of the following rare diseases or groups of rare diseases:

- Primary lipodystrophies (ORPHA90970):

* Congenital Generalized Lipodystrophy (CGL, also known as Berardinelli-Seip syndrome, ORPHA528).

* Familial Partial LipoDystrophy (FPLD), including Type 2 Familial Partial LipoDystrophy (FPLD2/Dunnigan syndrome, ORPHA2348) and other forms of FPLDs (ORPHA98305, -98,306, -79,083, -79,084, -280,356 and − 435,651).

* Acquired lipodystrophy, including Acquired Generalized Lipodystrophy (AGL, ORPHA79086) or Acquired Partial Lipodystrophy (APL, ORPHA98307), or HIV-related Lipodystrophy (either generalized or partial).

* Symmetric cervical adenolipomatosis (Launois-Bensaude disease, ORPHA2398).

- Other rare severe insulin-resistance syndromes (ORPHA181368), including patients with *INSR* mutations (type A insulin-resistance syndrome (ORPHA2297) and Rabson-Mendenhall syndrome (ORPHA769); with auto-antibodies against insulin receptor (type B insulin-resistance syndrome (ORPHA2298); or with premature ageing syndromes (ORPHA 139,033), including typical or atypical Werner syndrome (ORPHA902 and ORPHA79474).

Differential diagnosis were ruled out, in particular Cushing syndrome, acromegaly and other rare forms of diabetes [16].

### Statistics

Descriptive results are expressed as medians with interquartile ranges (IQRs) for continuous variables and percentages for categorical variables. A Welch two sample t-test was used for analysis of sex differences at age of referral. Data were analyzed with R Software (R Foundation for Statistical Computing, Vienna, Austria), using pvalue.io (a graphic user interface to the R statistical analysis software for scientific medical publications, https://www.pvalue.io/fr).

## Results

### General characteristics of the cohort (Fig. 1)

**Fig. 1:**
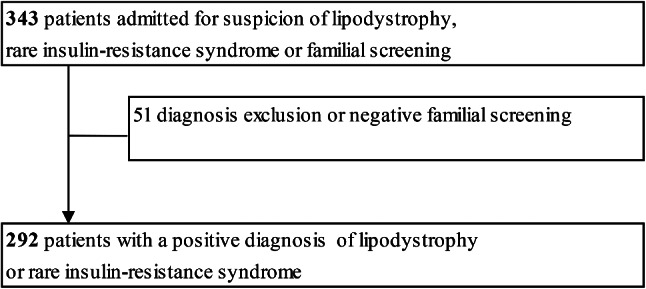
Flow Chart of the study

We studied an initial population of 343 patients, including 248 women, referred to the PRISIS coordinating reference center for lipodystrophy or rare insulin-resistance syndrome. Over half (55%) of the patients were living within 50 km of the reference center, 13% between 50 and 150 km, and 24% lived more than 150 km away, whereas 3% of the patients came from abroad.

Patients were mainly referred by a medical specialist (61%) or entered through the national network of rare disease centers (8%). Patients also consulted by their own initiative (8%) or through patient organizations (1%). Few patients were referred by their general practitioner (GP) (3%) or by a pediatrician (2%). For 17% of patients, the origin of referral was not specified. Following referral, 51 patients (14% of the cohort) were not considered affected by rare diseases covered by the reference center. The study thus analyzed a cohort of 292 patients, including 208 women (Fig. 1).

### Distribution of patients in the different diagnostic groups (Fig. 2A)

**Fig. 2:**
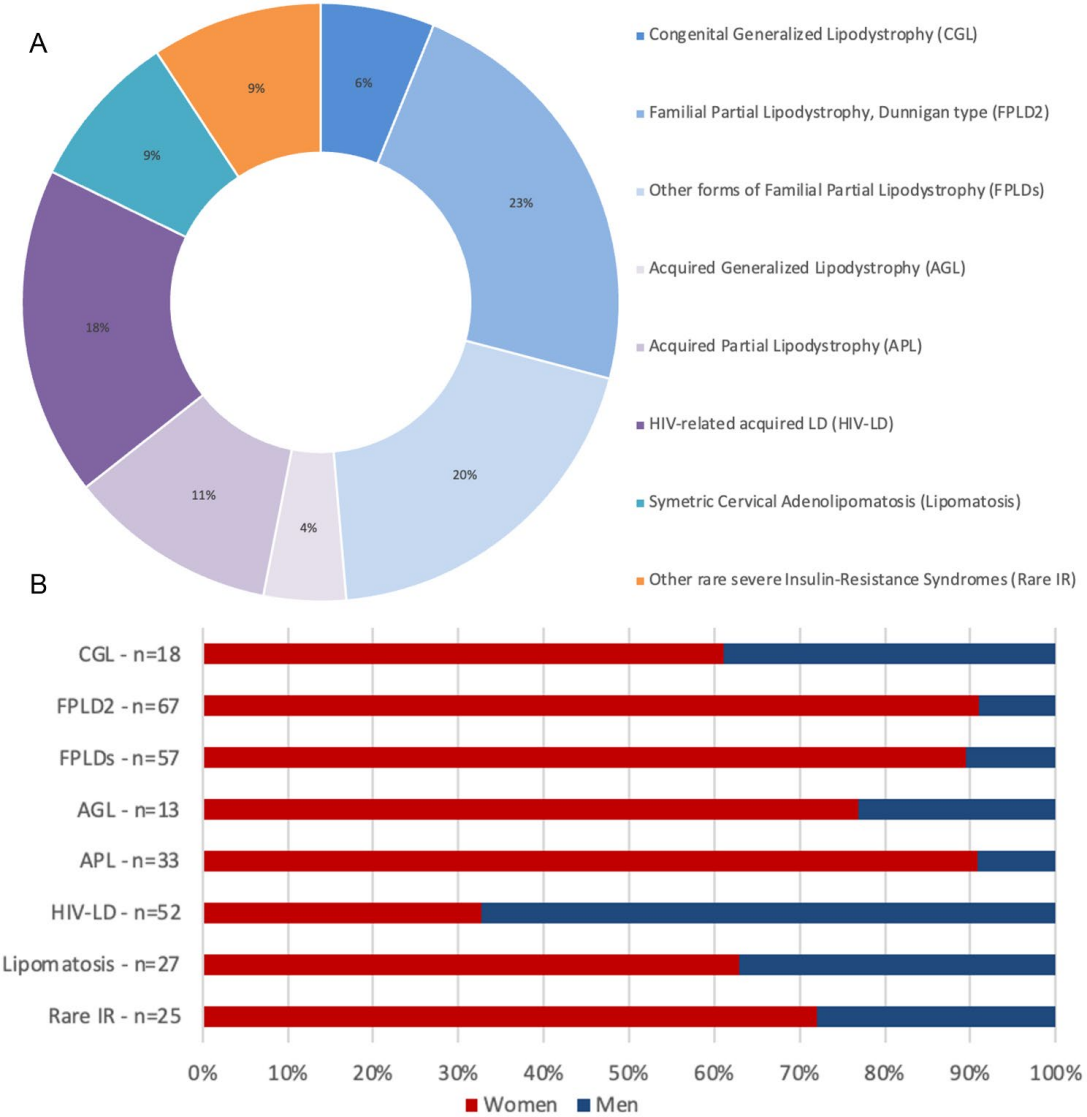
(**A**) Diagnostic group distribution in the cohort of 292 studied patients. Congenital Generalized Lipodystrophy (CGL)/Berardinelli-Seip syndrome: 18 patients (6% of the cohort); Type 2 Familial Partial LipoDystrophy (FPLD2/Dunnigan syndrome): 67 patients (23%); Other forms of FPLDs: 57 patients (20%); Acquired Generalized Lipodystrophy (AGL): 13 patients (4%); Acquired Partial Lipodystrophy (APL): 33 patients (11%); HIV-related Lipodystrophy (HIV-LD, either generalized or partial): 52 patients (18%); Symmetric cervical adenolipomatosis (Lipomatosis/Launois-Bensaude disease): 27 patients (9%); Other rare severe insulin-resistance syndromes (Rare IR): 25 patients (9%). (**B**) Gender distribution in each diagnostic group. Women represented the majority of patients (71% overall) in all but one diagnostic groups. The proportion of women represented 61% in CGL, 91% in FPLD2, 89% in other FPLDs, 63% in cervical symmetric lipomatosis, 76% in AGL, 90% in APL and 72% within the group of other rare severe insulin-resistance syndromes (Rare IR). It was only 19% in HIV-related lipodystrophies (HIV-LD)

- CGL (Berardinelli-Seip syndrome, ORPHA 528), mainly inherited in autosomal recessive manner, presents from birth or in early infancy as a generalized lipoatrophy with severe insulin-resistance [17]. CGL, confirmed by molecular analysis, was diagnosed in 18 patients (6% of the cohort). Pathogenic variants in *AGPAT2* (12/18: 67%) and *BSCL2* (2/18: 11%) genes account for the majority of cases. All patients had a confirmed diagnosis at referral.
- FPLD2 (ORPHA 2348) is an autosomal dominant syndrome, caused by pathogenic variants in the *LMNA* gene, encoding the nuclear proteins type A lamins. FPLD2 is characterized by a loss of subcutaneous adipose tissue from the trunk and limbs, which contrasts with fat accumulation in the upper body part and muscular hypertrophy developing from adolescence. The disease is progressively associated with metabolic complications linked to insulin-resistance, with glucose tolerance abnormalities leading to diabetes, dyslipidemia and liver steatosis [16]. FPLD2, confirmed by molecular analysis, represents the largest diagnostic group of our study, including 67 patients (23% of the whole cohort). The majority (63/67 patients, i.e. 94%) of patients had received a conclusive diagnosis at referral.
- Other forms of FPLDs (ORPHA 98,306), represented 57 patients (20% of the cohort), with 30 patients (53%) having a confirmed diagnosis at baseline. Patients were mainly affected with FPLD1 (Köbberling type; ORPHA 79,084) (*n* = 20 cases), a polygenic form of partial lipodystrophy [7]. Some patients presented with FPLD3 or FPLD4, due to *PPARG* (ORPHA 79,083) or *PLIN1* (ORPHA 280,356) pathogenic variants, respectively (*n* = 4 and 11 patients).
- AGL (ORPHA 79,086) can be associated with immune dysregulations targeting adipose tissue [18, 19], or remains of unknown origin. AGL affected 4% of our cohort i.e. 13 patients, 7 of them (53%) being diagnosed at baseline.
- APL/Barraquer-Simons syndrome (ORPHA 98,307) was diagnosed in 33 patients (11.3% of the cohort), with 22 of ascertained diagnosis at baseline (67%). Barraquer-Simons syndrome is characterized by bilateral, symmetrical lipoatrophy of cephalo-thoracic progression affecting the upper body (face, neck, arms, thorax). This disease, which affects mainly women, is, at least for some patients, of autoimmune origin and may be associated with membranoproliferative glomerulonephritis, low serum levels of complement component C3 and/or the presence of C3-nephritic factor [20].
- HIV-related lipodystrophy was described as the most frequent cause of generalized or partial acquired lipodystrophy [1–3], but its prevalence declined in parallel with the advances in HIV management and antiretroviral therapy. HIV-related lipodystrophies are secondary to multifactorial mechanisms resulting, among others, from the effects of antiretroviral agents and/or of HIV itself on adipose tissue [21]. Fifty-two patients (17.8% of our cohort) were admitted to our reference center for HIV-related acquired lipodystrophy.
- Symmetric cervical adenolipomatosis (multiple symmetric lipomatosis/Launois-Bensaude lipomatosis; ORPHA 2398), is a rare and heterogeneous disease with upper-body adipose tissue masses and/or lipodystrophy, either associated with alcoholism or of monogenic origin [22]. It was diagnosed in 27 patients from our center, at the time of referral in 14 of them.
- Finally, 25 patients (8.5% of the cohort) presented with other rare severe insulin-resistance syndromes, not elsewhere specified, including patients with genetic or autoimmune diseases affecting insulin receptor, or with premature ageing syndromes. Fifteen patients had an ascertained diagnosis at referral (60% of the group).

### Women were overrepresented in most diagnostic groups

Women represented the majority of patients (71% overall) in all diagnostic groups, except HIV-related lipodystrophies (19%). The proportion of women represented 61% in CGL, 91% in FPLD2, 89% in other FPLDs, 63% in cervical symmetric lipomatosis, 76% in AGL, 90% in APL and 72% within the group of other rare severe insulin-resistance syndromes (Fig. 2B). To note, in the FPLD2 subgroup, all men from our cohort were diagnosed following familial screening.

### Age at referral in the different diagnostic groups(Fig. 3)

**Fig. 3:**
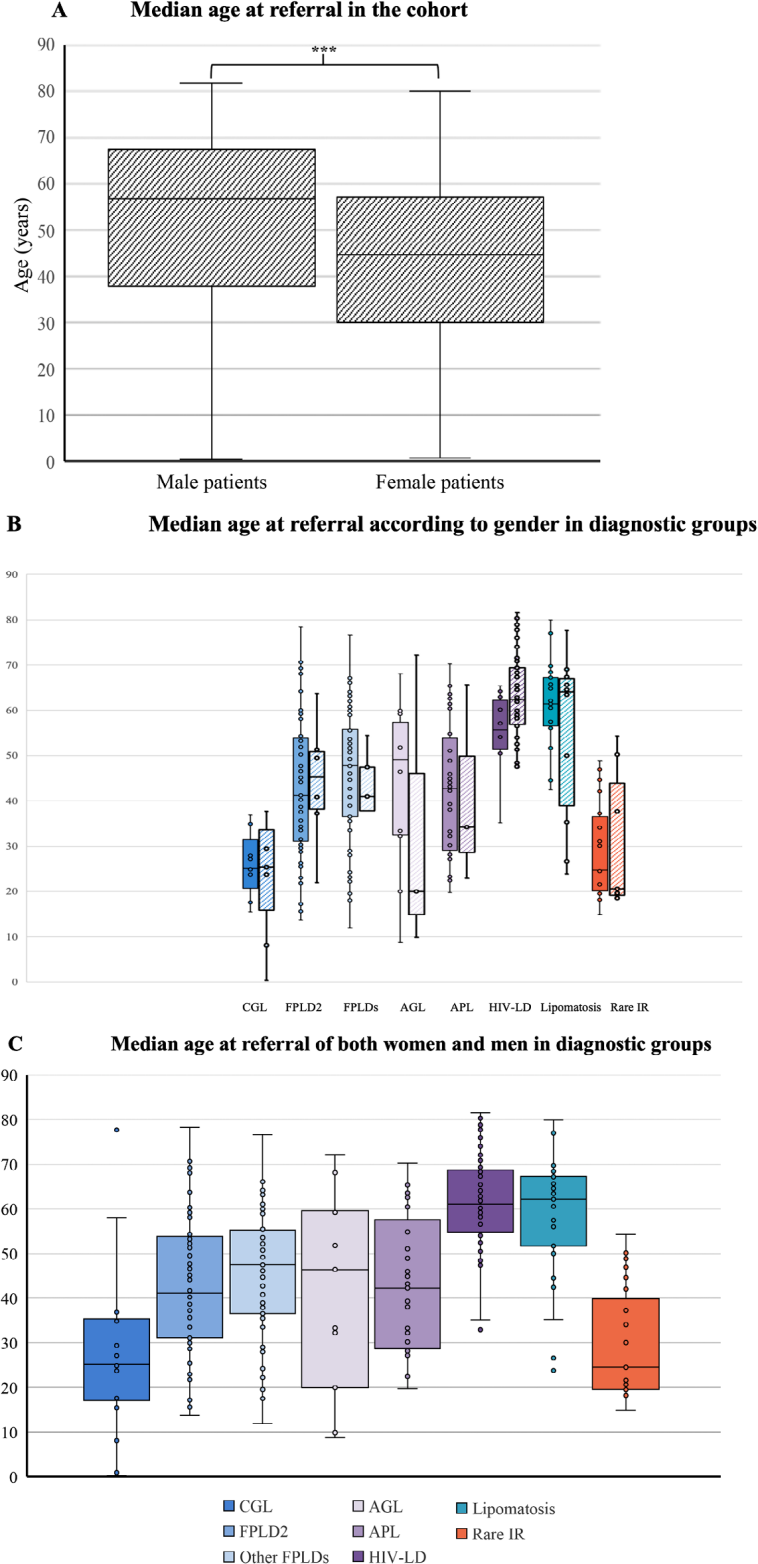
Median age at referral of patients in the cohort. (**A**) Boxplots of median age at referral in men and women of the whole cohort of patients. (**B**) Boxplots indicate the age at referral in the reference center for each disease group: Congenital Generalized Lipodystrophy/ Berardinelli-Seip syndrome: CGL - Familial Partial Lipodystrophy type 2/Dunnigan syndrome: FPLD2 - Other forms of Familial Partial Lipodystrophy: Other FPLDs - Acquired Generalized Lipodystrophy: AGL - Acquired Partial Lipodystrophy: APL - HIV-related lipodystrophy: HIV-LD - Symmetric cervical adenolipomatosis: Lipomatosis - Other rare severe insulin-resistance syndromes: Rare IR. In each diagnostic group, full coloured boxes depict women and dashed boxes depict men. (**C** )Boxplots of median age at referral in both men and women in each diagnostic group

The overall median age at referral, recorded for all patients, was 47.6 years [IQR: 31.3–60.3]. This age was significantly lower in women (44.7 [IQR: 30.1–56.9]), than in men (56.7 [IQR: 37.7–67.3]; *p* < 0.001) (Fig. 3A). However, within each diagnostic group, of limited size, we did not find any significant difference between the age at referral in men and women (Fig. 3B).

In our center dedicated to adult patients, the lowest age at referral was observed in patients with rare insulin-resistance syndromes (24.5 years [IQR: 19.6–37.7] and in patients with CGL (25.2 years [IQR: 17.1–33.5]), as expected in diseases of paediatric onset. Only 13 patients under the age of 18 (including nine girls) were referred at a median age of 13.7 years [IQR: 8–15]. Among them, five were diagnosed with CGL, three with FPLD2, two with other forms of FPLD, two with acquired generalized lipodystrophy, and one with a rare severe insulin-resistance syndrome.

The most advanced ages at referral were recorded in patients with HIV-related lipodystrophy and with symmetric cervical adenolipomatosis (61.1 years [IQR: 55.9–68.7] and 62.2 [IQR: 52.2–67.3], respectively). Patients from the other diagnosis groups were referred to our center at a median age ranging from 41.2 years [IQR: 31.2–53.5] for patients with FPLD2 to 47.5 [IQR: 36.5–54.7] in those with other FPLDs. Patients with non-HIV-related AGL and APL were referred at 46.4 [IQR: 19.9–59.2] and 42.3 [IQR: 28.7–58.8], respectively (Fig. 3C).

### Age at first clinical signs, and diagnostic and referral delays among different groups

The age at first sign of the disease and the age at diagnosis were recorded by the referent physician for 198 and 180 patients (68% and 62% of the whole cohort), respectively. When both items were available, i.e. in 161 patients (55%), we could measure the diagnostic delay. In the whole cohort, the median age at first clinical symptoms was 27.6 years [IQR: 16.8–42.0], and the diagnostic delay spanned 6.4 years [IQR: 1.3–19.5]. The referral delay, i.e. the delay between the first signs of the disease and referral to our center, available in 198 patients (68%), was 15.1 years [IQR: 6.2–27.2]. These indicators vary widely depending on different forms of lipodystrophy and insulin-resistance syndromes (Table 1).

**Table 1:** Main results for 292 patients and for each diagnostic group

	TOTAL	CGL	FPLD2	FPLDs	AGL	APL	HIV-LD	Cervical Lipomatosis	Rare IR
*Number of patients* *Sex ratio (W/M)*	292 215/77	18 11/7	67 61/6	57 51/6	13 10/3	33 30/3	52 17/35	27 17/10	25 18/7
**Median age** **at first signs** (years)	27.6 [IQR: 16.8–42.0]	0 [IQR: 0-9.7]	19.3 [IQR:14.4–27.8]	27.4 [IQR:19.0-37.6]	18.4 [IQR:8.4–29.7]	31.5 [IQR:16.3–43.9]	49.7 [IQR:41.3–54.8]	43.1 [IQR:30.3–54.5]	19.0 [IQR:16.7–25.8]
*No. of patients’ available data (%)*	198 (68%)	5 (27%)	58 (86%)	47 (82%)	10 (77%)	18 (55%)	27 (52%)	26 (96%)	7 (28%)
**Median age** **at diagnosis** (years)	41.1 [IQR: 24.3–53.8]	19.7 [IQR: 7,4-31.3]	32.8 [IQR:24.4–46.0]	43.9 [IQR:26.3–49.5]	30.8 [IQR:11.9–37.4]	34.2 [IQR:17.8–44.5]	53.6 [IQR:44.3–60.0]	58.8 [IQR:53.0-67.2]	22.5 [IQR:18.1–31.3]
*No. of patients’ available data (%)*	180 (62%)	11 (61%)	61 (91%)	32 (56%)	8 (62%)	15 (45%)	25 (48%)	20 (74%)	10 (40%)
**Median age** **at referral** (years)	47.6 [IQR: 31.3–60.3]	25.2 [IQR: 17.1–33.5]	41.2 [IQR: 31.2–53.5]	47.5 [IQR: 36.5–54.7]	46.4 [IQR: 19.9–59.2]	42.3 [IQR: 28.7–58.8]	61.1 [IQR: 55.9–68.7]	62.2 [IQR: 52.2–67.3]	24.5 [IQR: 19.6–37.7]
*No. of patients’ available data (%)*	292 (100%)	18 (100%)	67 (100%)	57 (100%)	13 (100%)	33 (100%)	52 (100%)	27 (100%)	25 (100%)
**Median diagnostic delay** (years)	6.4 [IQR: 1.4–19.3]	10.7 [IQR: 3.4–22.6]	10.5 [IQR: 1.8–27.0]	15.1 [IQR: 5.4–27.2]	1.08 [IQR: 0.4–2.2]	2.4 [IQR: 1.0-6.6]	1.7 [IQR: 0.3–6.5]	10.5 [IQR: 3.8–19.6]	7.9 [IQR: 3.3–11.2]
*No. of patients’ available data (%)*	161 (55%)	11 (61%)	55 (82%)	27 (47%)	8 (61%)	14 (42%)	24 (46%)	20 (74%)	6 (24%)
**Median** **referral delay** (years)	15.1 [IQR: 6.2–27.2]	25.1 [IQR: 21.6–36.7]	19.4 [IQR: 10.0-32.4]	16.8 [IQR: 7.3–25.3]	7.4 [IQR: 3.1–18.7]	6.5 [IQR: 2.7–18.9]	11.3 [IQR: 5.8–24.3]	12.0 [IQR: 4.7–21.7]	9.0 [IQR: 5.4–15.1]

Main results for 292 patients and for each diagnostic group. The total number and sex ratio of patients is shown for the cohort of 292 patients. For each group: Congenital Generalized Lipodystrophy (CGL/Berardinelli-Seip syndrome), Type 2 Familial Partial LipoDystrophy (FPLD2/Dunnigan syndrome), Other forms of FPLDs (FPLDs), Acquired Generalized Lipodystrophy (AGL), Acquired Partial Lipodystrophy (APL), HIV-related Lipodystrophy (HIV-LD), Symmetric cervical adenolipomatosis (Lipomatosis), Other rare severe insulin-resistance syndromes (Rare IR). Median age at first signs, at diagnosis, at referral, as well as median diagnostic delay and median referral delay are shown in each diagnostic group. IQR: InterQuartile Range

In patients with CGL, the mainly reported age at first sign of the disease was in infancy or early childhood. The age at diagnosis, available in 11/18 patients (61%), was below 12 years in 5 patients, but was delayed after the age of 35 years in 3 patients. The median diagnostic delay for CGL was 10.7 years [IQR: 3.45–22.6], and the referral delay to our adult reference center was 25.1 years [IQR: 21.6–36.7].

In patients with FPLD2 or other forms of FPLDs, the first clinical symptoms appeared mainly during young adulthood (median age of 19.3 years [IQR: 14.4–27.8] and 27.4 years [IQR: 19.0-37.6]), with a diagnostic delay of 10.5 years [IQR: 1.8–27.0] and 15.1 years [IQR: 5.4–27.2], and a referral delay as long as 19.4 years [IQR: 10.0-32.4] and 16.8 years [IQR: 7.3–25.3], respectively. In one case, the family genetic screening revealed a FPLD2 diagnosis almost five years before the onset of clinical symptoms (Fig. 4B).

The diagnostic delay was shorter in acquired forms of lipodystrophy (Table 1): 1.08 years [IQR: 0.4–2.2], 2.4 years [IQR: 1.0-6.6] and 1.7 years [IQR: 0.3–6.5] for AGL, APL and HIV-related lipodystrophy, respectively. Acquired lipodystrophies are characterized by a wide range of clinical onset, from a median 18.4 (AGL) to 31.5 (APL), and 49.7 years of age (HIV-related lipodystrophy). Patients with AGL and APL were referred to our center within a similar timeframe of 7.4 [IQR: 3.1–18.7] and 6.5 years [IQR: 2.7–18.9] after presenting signs, respectively. The referral delay of patients with HIV-related lipodystrophy was higher, as they are primarily followed by other specialists.

## Discussion

Diagnosis of rare diseases and referral to specialized centers are mandatory to provide affected patients with optimized medical management. Underdiagnosis of lipodystrophy and rare insulin-resistance syndromes is an important issue, due to the heterogeneous clinical presentation of patients, the lack of highly specific clinical signs and markers, and the lack of widespread medical knowledge regarding adipose tissue dysfunction and rare disorders of insulin sensitivity [6]. We took advantage of the data recorded by our coordinating reference center in the French National Rare Disease Database BNDMR [14] to focus on the time frame from first clinical symptoms to diagnosis and referral, in patients with lipodystrophy and rare insulin-resistance syndromes.

We considered eight groups of rare diseases encompassing lipodystrophy and severe insulin-resistance syndromes. We observed that the age at referral of patients reflects the natural history of the diagnostic groups, since it ranged from the lowest in patients with CGL and rare insulin-resistance syndromes, followed by patients with FPLDs; to the highest in patients with acquired lipodystrophies and symmetric cervical adenolipomatosis.

We show that FPLD2/Dunnigan syndrome, due to *LMNA* pathogenic variants, is the most frequent form of lipodystrophy in patients referred to our reference center, representing 23% of our recruitment, followed by other forms of FPLDs (20%). Although of genetic origin, the clinical expression of FPLDs progressively develops in late childhood or adulthood. CGL, a genetic form of lipodystrophy clinically expressed from birth or early childhood, only affects 6% of the referred patients, in line with the very rare prevalence of the disease. As expected, patients with CGL had a lower age at referral than patients with other lipodystrophy syndromes. However, patients with CGL could also be diagnosed in mid-childhood or even in adulthood. It is crucial to decrease the diagnosis delay of CGL, since patients may highly benefit from a specific orphan drug therapy with metreleptin [23]. The important delay from diagnosis to referral, as compared to the delay from first signs to diagnosis, illustrates the fact that reference networks should be promoted among caregivers in France.

Women are overrepresented in patients referred to our reference center, and their age at referral was overall significantly younger than that of men, although we did not show any significant difference within each diagnostic group, due to their limited size. Although FPLDs are monogenic diseases of autosomal inheritance and, as such, similarly affect both sexes, it was previously reported that Dunnigan syndrome is mostly diagnosed in female patients, due to several gender-related specificities of the disease. First, Dunnigan syndrome is more severe in women than in men [24–26]. Second, the lipodystrophic morphotype is more often socially concerning in women than in men [27, 28]. Third, the ovarian consequences of severe insulin-resistance syndromes, leading to hirsutism, anovulation and fertility disorders, also lead to a gender-specific recruitment of patients [29, 30]. The sex-ratio of patients referred to our coordinating reference center for CGL is more balanced, although a recent study reported that women with CGL also present earlier and more severe metabolic complications than men [17]. However, the female preponderance of FPLD2 diagnosis is higher in our study than in others [11, 25, 31–33]. This could result from the specific expertise of our endocrinology department on female fertility, which could favor the recruitment of women. In addition, male patients, known to display fewer symptoms, are probably followed outside of the reference center circuit.

The overall median time lag from first symptoms to diagnosis is 10.5 years, and the referral delay reaches a median 19.4 years in patients with Dunnigan syndrome. Although we could not infer from this study the probably multifactorial reasons that caused this important diagnostic delay; it is in line with overall data from the Erradiag study (https://dumas.ccsd.cnrs.fr/dumas-01932874) published by the French Rare Alliance in 2014 (https://www.alliance-maladies-rares.org/wp-content/uploads/2020/05/Erradiag-l-errance-diagnostic-dans-les-maladies-rares1.pdf). It states that, in France, 20% of patients with a rare disease, 5 years after their first symptoms, were still not referred to an academic hospital. At the EU level, the EURORDISCARE 1, 2 and 3 studies published by EURORDIS (https://www.eurordis.org/publications/the-voice-of-12000-patients), for 6, 8 and 16 rare diseases respectively, added more information about the care journey of patients with rare diseases. In 2006, the EURORDISCARE 2 study evaluated the diagnostic delay at 15 months to 28 years in 8 rare diseases (see page 43 in EURORDISCARE FULLBOOK;https://www.eurordis.org/wp-content/uploads/2009/12/EURORDISCARE_FULLBOOKr.pdf).

Continuous medical education is needed in the field of rare diseases to improve the knowledge of nonspecialized health professionals [34]. The knowledge about rare diseases was considered insufficient in a survey involving 111 GPs in Belgium [35], as was the use of the Orphanet website. A coordinated care pathway between GP and the reference centers in patients with rare diseases is required, as explored recently in the U.K [36]. In France, the effort of reference centers, driven by the national plan for rare diseases, to publish national guidelines easily available for GPs should improve the coordination of care pathways for each rare disease [14].

Our study has some limitations. Although analyzing the data from the coordinating reference center for lipodystrophy and other syndromes of insulin-resistance in France, it cannot depict the picture of care pathways of these rare diseases in the whole of France. The recruitment of our center, dedicated to adults, favors patients with insulin-resistance syndromes occurring after puberty. In addition to this selection bias, the impact of the COVID-19 pandemic on the care of patients with rare and/or undiagnosed diseases in France has been recently described [36]. Many patients are followed in other centers in France, particularly in one of the 26 other reference and competence centers of the PRISIS network throughout the country. Effort should be certainly undertaken to increase patient recruitment, including patients without severe disease expression, or complications, to provide patients with the best possible support during their care journey and prevent/delay complications associated with these chronic rare diseases. In addition, we were not able to retrieve information about the age at first symptoms and at diagnosis for all patients, showing that efforts should be made to enhance BaMaRa-BNDMR database implementation.

Finally, a substantial proportion of patients with lipodystrophy or insulin-resistance syndromes probably remain undiagnosed or diagnosed with type 2 diabetes and/or are simply not referred to a reference center. Patients currently undiagnosed, missed from this study, are susceptible to be recruited with higher diagnostic delay in the future, as our estimates seem to represent minimum values of the true median delays. In addition, we only recorded patients followed in our center after its official accreditation. Therefore, this study only represents a picture of the recruitment of our reference center during its first 5 year-period of official accreditation and should be completed by national or further prospective data.

A national multicentric study of patient self-reported data regarding first symptoms and subsequent diagnosis of genetic forms of lipodystrophy has been implemented [37]. It provides complementary information about patients’perspectives on the diagnostic and care journey in France. The Human Phenotype Ontology (HPO) tool, to be used in future versions of the BNDMR database, will certainly be very useful for deep phenotyping of patients.

## Conclusion

Following the objectives of the 3rd French rare diseases national plan, we aimed to describe the diagnostic and care pathways of patients with lipodystrophy and other rare syndromes of insulin-resistance, as recorded in the French National Rare Disease Database (BNDMR). Shortening the diagnostic and referral delays to focus on access to specialized care is crucial, in order to optimize medical care and genetic counselling, and avoid complications. A structured national implementation of the BNDMR database would benefit future extended surveys to describe the French cohort of patients with rare lipodystrophy and insulin-resistance syndromes followed in the rare disease PRISIS network. Data from the European Lipodystrophy registry, created by the European Consortium of Lipodystrophies (ECLip), will provide further information about the natural history of lipodystrophy and dedicated therapeutic pathways in a shared attempt to improve the care of patients with lipodystrophy at the European level [38].

## Data Availability

Since the data that support the findings of this study are not openly available due to reasons of sensitivity, the datasets used and/or analyzed during the current study are available from the corresponding author upon reasonable request. Data are located in controlled access data storage at the BNDMR (https://www.bndmr.fr/exploiter/donnees-bndmr/charte-bndmr/).

## Abbreviations

GP: General Practitioner
PRISIS: *Pathologies Rares de l’Insulino-Sensibilité et de l’Insulino-Sécrétion*”:Reference Center for Rare Diseases of Insulin Secretion and Insulin Sensitivity
BNDMR: *Banque Nationale de Données Maladies Rares*”:French National Rare Disease Database
